# Biological properties and potential pathogenic mechanisms of oral bacterial-derived extracellular vesicles in oral and systemic diseases

**DOI:** 10.20517/evcna.2026.13

**Published:** 2026-05-29

**Authors:** Xumeng Du, Shiyin Xu, Xiaolei Zhou, Huanhuan Zhou, Biyu Jin, Zhao Peng, Andreas K. Nüssler, Liegang Liu, Jiuling Chen, Wei Yang

**Affiliations:** ^1^Department of Nutrition and Food Hygiene, Hubei Key Laboratory of Food Nutrition and Safety, Tongji Medical College, Huazhong University of Science and Technology, Wuhan 430030, Hubei, China.; ^2^Department of Nutrition and Food Hygiene and MOE Key Lab of Environment and Health, School of Public Health, Tongji Medical College, Huazhong University of Science and Technology, Wuhan 430030, Hubei, China.; ^3^NHC Specialty Laboratory of Food Safety Risk Assessment and Standard Development, Wuhan 430030, Hubei, China.; ^4^Department of Traumatology, BG Trauma Center, University of Tübingen, Tübingen 72076, Germany.; ^5^Department of Thoracic Surgery, Union Hospital, Tongji Medical College, Huazhong University of Science and Technology, Wuhan 430022, Hubei, China.

**Keywords:** Oral bacterial extracellular vesicles, oral diseases, systemic diseases, pathogenesis, oral-systemic axis

## Abstract

This review summarizes the biological characteristics of oral bacterial extracellular vesicles (O-BEVs) and their proposed roles in disease pathogenesis. As nanosized lipid bilayer structures secreted by oral bacteria, O-BEVs encapsulate virulence factors, nucleic acids, and metabolites, enabling them to traverse biological barriers and modulate host cellular functions. Studies suggest that O-BEVs are involved in the progression of oral diseases, including periodontitis, dental caries, and oral cancer through mechanisms such as immune activation, tissue destruction, and cellular behavior modulation. Furthermore, O-BEVs can enter the systemic circulation. Through synergistic interactions among their protein, lipid, and nucleic acid components, O-BEVs activate inflammatory pathways and compromise barrier integrity, thereby contributing to the pathogenesis of systemic diseases, including Alzheimer’s disease, atherosclerosis, diabetes mellitus, rheumatoid arthritis, adverse pregnancy outcomes, and osteoporosis. However, most current evidence derives from preclinical (*in vitro* and animal) studies, while direct clinical evidence linking O-BEVs to human diseases remains limited, and causal relationships have not been firmly established. Understanding the mechanisms of O-BEVs may enable targeted diagnostic approches, therapies, and prevention strategies along the “oral-systemic axis”, with important clinical and public health implications.

## INTRODUCTION

The oral cavity, serving as one of largest microbial reservoir in the human body, harbors a unique microecological environment^[1]^. Its complex anatomical structures, such as gingival sulci and the dorsum of the tongue, together with favorable temperature, humidity, and abundant nutrients, provide ideal conditions for microbial colonization and proliferation^[2]^. The oral microbiota consists primarily of three main types: bacteria, fungi, and viruses, with bacteria representing the most abundant and diverse group^[3]^. It is estimated that the total number of oral bacteria exceeds trillions, encompassing around 1,000 distinct bacterial species^[4]^. These bacteria exhibit intricate interdependencies and competitive interactions, forming a dynamic equilibrium through symbiotic, competitive, and antagonistic relationships that collectively promote the stability and health of the oral ecosystem^[5]^.

However, the oral cavity is not a hermetically sealed system. When the oral mucosal barrier is compromised by ulcerations or daily activities such as chewing, oral bacteria and their metabolic products can enter the bloodstream, causing transient bacteremia^[6,7]^. Furthermore, inflammatory stimuli increase the permeability of the periodontal pocket epithelium, facilitating the penetration of oral bacteria and their metabolites into the microvasculature of periodontal tissues and subsequently their entry into systemic circulation^[8]^. Accumulating evidence has demonstrated associations between the oral microbiota and a range of systemic disorders, namely Alzheimer’s disease, atherosclerosis, and rheumatoid arthritis (RA), a concept collectively termed the “oral-systemic axis” effect^[2,7,9,10]^. Nevertheless, conventional paradigms focusing on the translocation of live bacteria or the diffusion of soluble toxins may not adequately explain the detection of oral bacterial components in distant organs, such as cerebral plaques, atherosclerotic lesions, and synovial fluid, in individuals without overt clinical signs of bacteremia^[11]^. This discrepancy suggests the potential existence of a more efficient and less readily detectable vehicle for microbial-host interactions beyond viable bacteria.

Importantly, the discovery of bacterial extracellular vesicles (BEVs) may help explain this paradox. BEVs are nanoscale (20-400 nm) lipid-bilayer vesicles that are actively secreted by bacteria and encapsulate a complex molecular cargo of membrane lipids, virulence factors, nucleic acids, and metabolites^[12-14]^. Their bilayer structure not only enables the traversal of biological barriers, but also protects the payload from enzymatic degradation, establishing BEVs as stealthy messengers in microbiota-host interactions^[15]^. Similarly, oral bacterial extracellular vesicles (O-BEVs) efficiently package and deliver bioactive molecules such as lipopolysaccharide (LPS), proteases and other immunomodulatory molecules. Upon binding to specific surface receptors, O-BEVs trigger intracellular signaling pathways that modulate cellular physiology and behavior in recipient cells^[16]^. Under normal physiological conditions, O-BEVs participate in intra- and inter-species bacterial communication, helping bacteria sense environmental changes, transmitting quorum-sensing signals, and regulating key activities including proliferation, metabolism, and biofilm formation^[17]^. In terms of pathogenicity, O-BEVs play multifaceted roles in host-pathogen interactions. As enriched vehicles for pathogen-associated molecular patterns (PAMPs) such as LPS and lipoproteins, O-BEVs bind to host pattern recognition receptors, thereby activating innate immune response and driving chronic inflammatory responses^[18,19]^. Simultaneously, O-BEVs deliver an arsenal of virulence factors that directly modify critical host target proteins. These modifications may expose cryptic self-epitopes, thereby breaking immune tolerance and triggering autoantibody generation, potentially contributing to autoimmune pathologies^[20]^. Furthermore, O-BEVs employ “molecular mimicry” by displaying epitopes that mirror those on host-derived extracellular vesicles. This camouflage not only facilitates immune evasion, but also enables enhanced targeting toward specific host cells,, ensuring the precise delivery of their pathogenic cargo to recipient host cells^[21]^.

In summary, in-depth investigation into the biological properties of O-BEVs and their pathogenic mechanisms in systemic diseases may facilitate the utilization of these vesicles in the prevention and treatment of such conditions. Therefore, this review focuses on the biological characteristics of O-BEVs, systematically summarizes their multifaceted regulatory mechanisms in systemic diseases, and discusses future challenges. We also suggest that this work will contribute to developing more effective oral health interventions aimed at maintaining oral microbial homeostasis and reducing the pathogenicity of oral bacteria and their vesicles, thereby potentially reducing the risk of systemic diseases and providing a theoretical basis for precision medicine targeting the “oral-systemic axis”.

## OVERVIEW OF O-BEVS

### Classification and biogenesis

**Figure 1:**
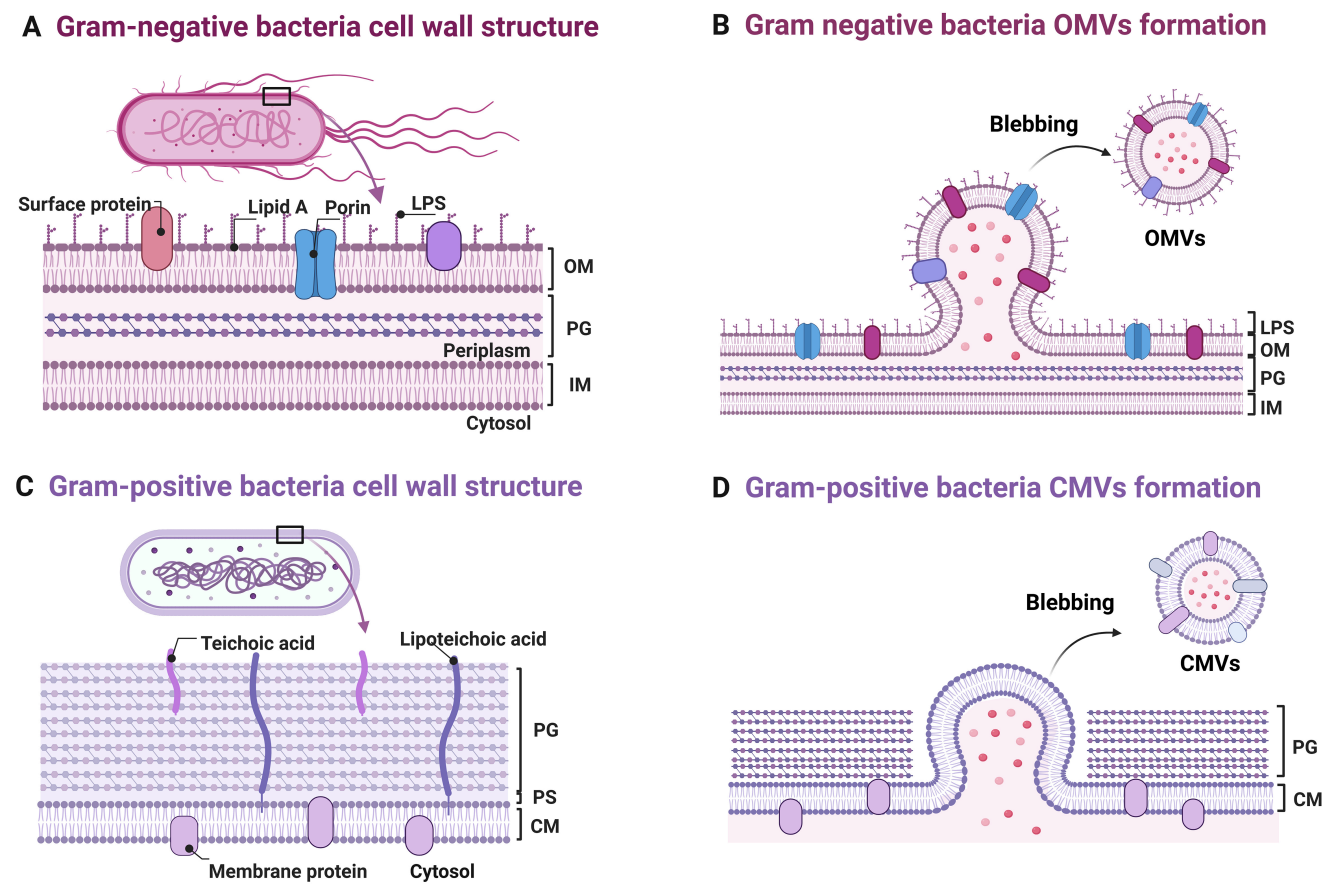
Schematic representation of bacterial membrane vesicle biogenesis and cell wall architecture. Multilayered cell wall structure comprising, from the outermost to innermost, LPS/OM, a PG layer, and an IM. The OM undergoes local evagination to form OMVs that encapsulate LPS, porins, and surface proteins; (B) Magnified view of OMV budding: Asymmetry in OM lipid composition and remodeling of lipid A drive membrane curvature and vesicle scission from the cell envelope; (C) Gram-positive cell envelope architecture consisting of a thick PG layer that directly encloses the CM, with teichoic acids and lipoteichoic acids interspersed throughout; (D) CM evagination leads to CMV formation, wherein membrane proteins and cytosolic cargo are encapsulated. These vesicles are released extracellularly via a yet-to-be-elucidated mechanism that likely traverse the thick PG layer. Note: Other vesicle subtypes (e.g., OIMVs, eOMVs, nanotubules) have been reported in bacteria but remain to be characterized in oral species; accordingly, they are not depicted in this schematic. Created in BioRender. Xumeng, D. (2026) https://BioRender.com/8w62rdx. LPS: Lipopolysaccharide; OM: outer membrane; PG: peptidoglycan; IM: inner membrane; OMVs: outer membrane vesicles; CM: cytoplasmic membrane; CMV: cytoplasmic membrane vesicle; OIMVs: outer-inner membrane vesicles; eOMVs: explosive outer membrane vesicles; PS: periplasmic space.

As important mediators of bacterial virulence, O-BEVs are generated through distinct biogenesis pathways influence by bacterial cell envelope architecture^[22]^. Gram-negative bacteria possess an outer membrane (OM), which serves as the direct source for vesicle budding, an inner membrane (IM), a periplasmic space (PS), and a thin peptidoglycan (PG) layer^[23]^. In contrast, Gram-positive bacteria are characterized by a thick, multi-layered PG cell wall that encases the cytoplasmic membrane (CM) and presents a physical barrier to vesicle release^[12]^. These structural distinctions give rise to several types of vesicles, including outer membrane vesicles (OMVs) from Gram-negative species, cytoplasmic membrane vesicles (CMVs) from Gram-positive species, as well as other less common or condition-dependent forms such as outer-inner membrane vesicles (OIMVs), explosive OMVs, and nanotubules^[24,25]^. Among these, OMVs and CMVs represent the most abundant and best-characterized vesicle types among O-BEVs, and they are considered important mediators of bacterial virulence in periodontitis and associated systemic diseases^[26,27]^. Therefore, the subsequent sections describe the biogenesis processes of OMVs and CMVs [Figure 1]^[28]^.

In Gram-negative oral bacteria such as *Porphyromonas gingivalis* (*P. gingivalis*), *Tannerella forsythia* (*T. forsythia*), *Aggregatibacter actinomycetemcomitans* (*A. actinomycetemcomitans*), *Fusobacterium nucleatum* (*F. nucleatum*), and *Treponema denticola* (*T. denticola*), OMVs are secreted with a typical diameter of 20-250 nm^[12]^. These OMVs are enriched in surface-associated virulence factors, capable of inducing inflammatory responses and damaging host cells, thereby contributing to the onset or progression of oral diseases and systemic conditions^[29]^. The biogenesis of OMVs is a multi-step process initiated by a dynamic imbalance between the OM and the PS, involving membrane remodeling, budding dynamics, and fission^[15]^. Key factors promoting OM protrusion include the asymmetric distribution of OM lipids, the accumulation of hydrophobic molecules or protein complexes, and the remodeling of the PG layer, which may involve the dissociation of Braun’s lipoprotein^[30]^. Subsequently, the OM undergoes reorganization, with phospholipids and LPSs rearranging to form spherical vesicles. Endolysins may further promote this process by locally degrading the PG layer, thereby facilitating OM budding^[31]^.

In contrast, CMVs derived from Gram-positive oral bacteria - including *Filifactor alocis* (*F. alocis*), *Streptococcus gordonii* (*S. gordonii*), *Stomatobaculum longum* (*S. longum*), *Streptococcus mutans* (*S. mutans*), *Streptococcus sanguinis* (*S. sanguinis*), and *Streptococcus oralis* (*S. oralis*) - must overcome the physical barrier of the thick cell wall during biogenesis^[15]^. These CMVs are secreted with a typical diameter of 20-400 nm^[12]^. This process is typically initiated by the localized degradation of the PG layer by endolysins, creating pores that expose the CM as budding sites. Subsequently, the membrane bulges outward through these pores, accompanied by the reorganization of lipids and proteins^[12]^. Membrane-associated and curvature-stabilizing proteins interact with the lipid bilayer to facilitate the formation of sealed vesicular structures^[30]^. During membrane lipid reorganization, specific lipids such as cardiolipin accumulate at budding sites, promoting vesicle formation and sealing by increasing membrane curvature and reducing fluidity. Meanwhile, cytoskeletal-like proteins and other curvature-associated proteins interact with lipids to regulate membrane shape and stability, facilitating CMVs biogenesis^[25]^. For vesicle escape, CMVs traverse the cell wall through pores remaining after endolysin degradation or potentially through phage-like channel proteins^[15,25]^.

### Composition and synergistic mechanisms

#### Protein components

The protein components of O-BEVs serve as major functional mediators of their pathogenicity and immunomodulatory effects. These vesicular proteins facilitate disease progression through multiple synergistic mechanisms, including direct cytotoxicity, disruption of host barriers, and dysregulation of immune responses.

The OMVs derived from *P. gingivalis* (*P. g-*OMVs) are enriched with gingipains, including the lysine-specific protease (Kgp) and the arginine-specific proteases (RgpA, RgpB). These proteases directly degrade host tight junction proteins, thereby disrupting the gingival epithelial barrier. Simultaneously, they activate host matrix metalloproteinases (MMPs), accelerating periodontal tissue destruction and driving periodontitis progression^[32]^. Furthermore, the peptidylarginine deiminase (PPAD) carried by *P. g-*OMVs converts host proteins (e.g., fibrinogen) into citrullinated forms, generating autoantigens and inducing the formation of anti-citrullinated protein antibodies (ACPAs), suggesting a potential link to autoimmune diseases like RA^[33]^. OMVs produced by *F. nucleatum* (*F. n-*OMVs) carry the Fusobacterium adhesin A (FadA), which interacts with host E-cadherin and activates the β-catenin signaling cascade, thereby promoting periodontitis as well as colorectal cancer cell proliferation and metastasis^[34,35]^. The OMVs from *A. actinomycetemcomitans* (*A. a-*OMVs) are enriched in leukotoxin (LtxA), which selectively targets host immune cells^[36]^. Additionally, these vesicles deliver the cytolethal distending toxin (CDT) to HeLa cells and human gingival fibroblasts (HGFs), inducing characteristic cell cycle arrest and cytoplasmic distension^[37]^. The OMVs from *T. denticola* (*T. d-*OMVs) carry adhesins and serine proteases essential for attachment to and degradation of host cells and mammalian extracellular matrix proteins^[38]^. *T. denticola* expresses a major surface glycoprotein termed Msp, which is associated with microbial adhesion, immune modulation, and pore formation^[39]^. The OMVs derived from *T. forsythia* (*T. f-*OMVs) harbor various virulence factors, including the leucine-rich repeat family protein BspA, which acts as a Toll-like receptor 2 (TLR2) agonist. Additioanl non-TLR2 agonist factors include sialidase and GroEL^[40]^.

Given the significant differences in the composition and function of vesicles from various bacterial species, further studies on vesicles produced by *F. alocis* and the cariogenic pathogen *S. mutans* have revealed species-specific virulence factors associated with these two species. The CMVs derived from *F. alocis* (*F. a*-CMVs) were reported to contain 28 proteins identified by proteomic analysis, including lipoproteins, autolysins, and the *F. alocis* complement inhibitor (FACIN)^[41]^. The CMVs derived from *S. mutans* (*S. m*-CMVs) carry key virulence factors, including glucosyltransferases (Gtfs) such as GtfB and GtfC^[42]^.

#### Lipid components

The LPS of O-BEVs plays dual roles in inflammatory activation and membrane stabilization, directly influencing their pathogenicity and therapeutic potential. LPS is abundant in OMVs and consists of lipid A, a core oligosaccharide, and an O-antigen polysaccharide chain^[24]^. Typically, lipid A structures activate innate immune responses via the TLR4/nuclear factor-κB (NF-κB) pathway, although LPS exhibits functional heterogeneity among different bacterial strains^[43]^. Lipidomic data have revealed that the levels of phosphatidylglycerol and stearic acid - key regulators of membrane fluidity and rigidity - are significantly higher in BEVs than in the OM of parent cells^[44]^. Cardiolipin is reported to be enriched in CMVs; it not only maintains vesicle structural stability via hydrophobic microdomains but also acts as a TLR4 ligand to activate the inflammasome, amplifying inflammatory signals and promoting pathological progression^[15]^. Of note, *P. gingivalis* LPS contains penta-acylated lipid A that serves as a TLR4 antagonist. Such structural characteristics allow the bacterium to escape host immune defense, thereby illustrating the functional diversity of LPS^[45]^. This lipid-mediated dual functionality - being both immune-evasive and membrane stabilizing - provides additional insight into disease pathogenesis and may inform precision therapeutic strategies that modulate lipid composition to balance the pathogenic and therapeutic potential of O-BEVs.

#### Nucleic acid components

The nucleic acid components of O-BEVs contribute to bacterial adaptive evolution and disease development through cross-species gene transfer and modulation of host epigenetic regulation. The *P. g*-OMVs, *T. d*-OMVs, and *T. f*-OMVs contain DNA and RNA that can stimulate TLR7, TLR8, and TLR9. Additionally, eDNA is found on the surface of these vesicles, where it forms an eDNA/OMVs network. This network may facilitate pathogen capture of nutrients within polymicrobial biofilms^[46]^. The *A. a*-OMVs contain eRNA, which is shielded from degradation in body fluids by encapsulation within OMVs and can be transferred into host cells. Once inside, it may integrate into host RNA-induced silencing complexes, thereby modulating the expression of host target transcripts^[47]^. Moreover, msRNAs are present in *A. a*-OMVs, *T. d*-OMVs, and *P. g*-OMVs*.* These vesicles can stably transfer the msRNAs into host cells, thereby regulating immune responses and apoptosis^[48]^. Furthermore, the nucleic acid-mediated horizontal gene transfer not only contributes to bacterial evolution and biofilm formation, but also may contribute to the dissemination ofantibiotic resistance.

#### Synergistic mechanisms of O-BEV components

The pathogenicity and therapeutic potential of O-BEVs stem from synergy among their proteins, lipids, and nucleic acid components, which together form a dynamic regulatory network that remodels the host microenvironment. In the context of pathogenic mechanisms, lipid-protein synergy significantly drives inflammatory cascades. For instance, after *P. gingivalis*-derived LPS activates the NF-κB pathway via TLR4, the PPAD within OMVs further amplifies inflammatory signaling, leading to excessive secretion of interleukin-6 (IL-6) and tumor necrosis factor alpha (TNF-α), exacerbating periodontitis and systemic inflammation^[32]^. The *F. n*-OMVs exert synergistic effects through the FadA protein and LPS to activate the Wnt/β-catenin pathway, promoting epithelial-mesenchymal transition (EMT) in colorectal cancer cells^[34]^. Interactions between vesicular metabolites and host signaling pathways are also important: succinate delivered by O-BEVs induces metabolic reprogramming (enhanced glycolysis) in macrophages via succinate receptor 1 (SUCNR1)^[49]^. Moreover, the inherent tissue-homing capability of O-BEVs could be exploited for targeted drug delivery, transforming these pathogenic vehicles into therapeutic carriers^[50]^. These synergistic mechanisms help to illustrate the potential involvement of O-BEVs in chronic inflammation, cancer, and other diseases, and may offer insights for developing component-based therapeutic strategies.

## PATHOGENIC MECHANISMS OF O-BEVS IN DIFFERENT DISEASES

### Oral diseases

#### Periodontitis

Recent advances have highlighted that extracellular vesicles both participate in periodontal pathogenesis and hold therapeutic potential for tissue regeneration^[51]^. As detailed in Table 1, O-BEVs from multiple oral pathogens contribute to periodontitis through two major mechanistic themes: (i) activation of innate immune receptors leading to pro-inflammatory cytokine release; and (ii) direct degradation of host tissue barriers.

**Table 1:** The role of O-BEVs in oral diseases

**Diseases**	**O-BEVs source**	**Key effector molecules**	**Core effect**	**Key pathways/targets**	**Ref.**
Periodontitis	*P. gingivalis* OMVs	Gingipains, LPS	Pro-inflammatory	MAPK/STING/NF-κB; ↑IL-6/IL-8	[46,53]
	*F. alocis* CMVs	Lipoproteins, FACIN	Pro-inflammatory	↑IL-6/IL-8/TNF-α, CCL1/2, G-CSF in keratinocytes and monocytes	[41]
	*T. forsythia* OMVs	BspA, sialidase, GroEL	Pro-inflammatory	↑IL-6/IL-8/MCP-1 in U937/hPDLCs	[54]
	*T. denticola* OMVs	Msp, chymotrypsin-like protease	Barrier disruption	Degrades intercellular adhesion proteins	[55]
	*F. nucleatum* OMVs	FadA, LPS	Pro-inflammatory, bone loss	NLRP3/NF-κB; M1 polarization; ↑IL-1β/IL-18	[35,57]
Dental caries	*S. mutans* CMVs	GtfB, GtfC	Biofilm formation, cross-kingdom interaction	EPS synthesis; quorum-sensing; ↓*S. gordonii*, ↑*C. albicans*	[58-61]
Oral cancer	*P. gingivalis* OMVs	sRNA23392, gingipains	Pro-tumorigenic	↓TNFSF15/ASPM; inhibits cGAS-STING	[63-65]
	*S. longus* CMVs	Unknown	Pro-tumorigenic	BRCA1/EXO1/TP53BP1 pathway	[66]
	*F. nucleatum* OMVs	FadA	EMT, metastasis	↓E-cadherin, ↑vimentin	[67]
	*A. actinomycetemcomitans* OMVs	LtxA, CDT	Anti-tumorigenic	Reduces proliferation; induces apoptosis (cell line-dependent)	[68,69]

*P. gingivalis*: *Porphyromonas gingivalis*; O-BEVs: oral bacterial extracellular vesicles; OMVs: outer membrane vesicles; LPS: lipopolysaccharide; MAPK: mitogen-activated protein kinase; STING: stimulator of interferon genes; NF-κB: nuclear factor-κB; IL-6: interleukin-6; IL-8: interleukin-8; *F. alocis*: *Filifactor alocis*; CMVs: cytoplasmic membrane vesicles; FACIN: *Filifactor alocis* complement inhibitor; TNF-α: tumor necrosis factor alpha; CCL1/2: C-C motif chemokine ligand 1/2; G-CSF: granulocyte colony-stimulating factor; *T. forsythia*: *Tannerella forsythia*; MCP-1: monocyte chemoattractant protein-1; hPDLCs: human periodontal ligament cells; *T. denticola*: *Treponema denticola*; Msp: major surface protein; *F. nucleatum*: *Fusobacterium nucleatum*; FadA: Fusobacterium adhesin A; NLRP3: NOD-, LRR- and pyrin domain-containing protein 3; IL-1β: interleukin-1 beta; IL-18: interleukin-18; *S. mutans*: *Streptococcus mutans*; Gtf: glucosyltransferase; EPS: extracellular polymeric substance; *S. gordonii*: *Streptococcus gordonii*; *C. albicans*: *Candida albicans*; sRNA: small RNA; ASPM: abnormal spindle microtubule assembly; cGAS: cyclic GMP-AMP synthase; *S. longus*: *Streptococcus longus*; BRCA1: breast cancer type 1 susceptibility protein; EXO1: exonuclease 1; TP53BP1: tumor protein P53 binding protein 1; EMT: epithelial-mesenchymal transition; *A. actinomycetemcomitans*: *Aggregatibacter actinomycetemcomitans*; LtxA: leukotoxin; CDT: cytolethal distending toxin.

In multiple O-BEV types, the activation of TLRs and NOD-like receptors (NLRs) represents a shared initiating event^[46,52]^. *In vitro* studies have shown that *P. g-*OMVs engage the mitogen-activated protein kinase (MAPK), stimulator of interferon genes (STING), and NF-κB pathways in gingival epithelial cells, upregulating IL-6 and interleukin-8 (IL-8)^[53]^. Similarly, *F. alocis* CMVs induce a broad panel of cytokines [IL-6, IL-8, TNF-α, granulocyte colony-stimulating factor (G-CSF), granulocyte-macrophage colony-stimulating factor (GM-CSF), C-C motif chemokine ligand 1/2 (CCL1/2), macrophage inflammatory protein-1 (MIP-1)] in both oral keratinocytes and monocytes^[41]^. Meanwhile, *T. f*-OMVs stimulate IL-6, IL-8, and monocyte chemoattractant protein-1 (MCP-1) release from periodontal ligament fibroblasts^[54]^. Notably, although these O-BEVs originate from different bacterial species (Gram-negative *vs.* Gram-positive, red complex *vs.* orange complex), they converge on similar pro-inflammatory cytokine profiles, suggesting that distinct upstream receptors funnel into common downstream signaling cascades.

In contrast to the shared inflammatory pathways, some O-BEVs exhibit unique mechanisms that directly compromise tissue integrity. The *T. d*-OMVs degrade intercellular adhesion proteins via the pore-forming Msp and chymotrypsin-like protease, thereby disrupting the epithelial barrier^[55]^. The *F. n*-OMVs not only promote M1 macrophage polarization, enhancing the inflammatory microenvironment and enhancing their cytotoxicity toward mouse gingival fibroblasts (MGFs)^[56]^, but also enter human periodontal ligament stem cells (hPDLSCs) via endocytosis, where the vesicles activate the NOD-, LRR- and pyrin domain-containing protein 3 (NLRP3) inflammasome and NF-κB (p65) pathway, upregulate interleukin-1 beta (IL-1β)/interleukin-18 (IL-18), and inhibit cell mineralization capacity - effects that may contribute to alveolar bone resorption^[35,57]^.

While the above findings are mechanistically informative, several limitations should be noted. Most studies are *in vitro* experiments using immortalized cell lines, often at O-BEVs concentrations whose physiological relevance has not been firmly established. Direct *in vivo* evidence linking specific O-BEVs subtypes to periodontitis progression in humans is limited, and the relative contribution of OMVs *vs.* CMVs *vs.* the parental bacteria themselves remains poorly defined. Future studies employing animal models with clinically relevant O-BEVs doses and longitudinal human cohort studies are needed to validate these mechanistic insights.

#### Dental caries

*S. mutans* is a major cariogenic bacterium, and its secreted CMVs (*S.m*-CMVs) have been implicated in bacterial colonization, adhesion, and invasion on tooth surface^[58]^. As summarized in Table 1, the cariogenic potential of *S.m*-CMVs is largely associated with glucosyltransferase (GTF)-mediated extracellular polymeric substance (EPS) synthesis.

*In vitro* studies have shown that GtfB and GtfC - major protein components of these vesicles - utilize sucrose to synthesize EPS, thereby enhancing *S. mutans* biofilm formation^[59]^. Among these, GtfC plays a dual role, not only mediating CMVs aggregation and biofilm formation, but also acting as an important antigen to elicit specific antibody production in mucosal immunity^[42]^. Furthermore, *S. m*-CMVs interact with other oral microorganisms, influencing their growth and metabolism. The CMVs from GTF-deficient *S. mutans* mutants inhibit *S. gordonii* biofilm formation without affecting planktonic growth^[60]^, while *S. m*-CMVs enhance sucrose metabolism and EPS production in *Candida albicans* (*C. albicans*), promoting cross-kingdom biofilm development^[61]^. This dual regulatory capacity - promoting a cariogenic microorganism while suppressing competing commensals - highlights the complex role of CMVs in shaping the oral biofilm community.

#### Oral cancer

Oral squamous cell carcinoma (OSCC) is the most common type of malignant tumor of the oral and maxillofacial region^[62]^. As detailed in Table 1, O-BEVs from multiple bacterial species exert divergent effects on OSCC, ranging from pro-tumorigenic to anti-tumorigenic.

Studies have shown that *P. g*-OMVs promote OSCC proliferation, migration, and immune evasion through multiple mechanisms: (i) delivering the small RNA sRNA23392, which targets host mRNAs and reduces their stability^[63]^; (ii) downregulating tumor suppressors (TNFSF15, ZNF292, ATRX) and cancer-related genes (ASPM, KIF20B) in CAL27 and HN6 cells^[64]^; and (iii) suppressing the cGAS-STING pathway, thereby impairing IFN-β production and reducing recruitment of natural killer cells and dendritic cells^[65]^. Similarly, *S. longus*-derived CMVs have been reported to promote OSCC malignancy via the BRCA1/EXO1/TP53BP1 pathway^[66]^, and *F. n*-OMVs induce EMT and autophagy, facilitating lung metastasis in mouse models^[67]^. In contrast, *A. a*-OMVs have been shown to reduce proliferation and increase apoptosis in certain OSCC cell lines (SCC-24A, HSC-3)^[68,69]^. This dichotomy - pro-tumorigenic *P. g*-OMVs *vs.* anti-tumorigenic *A. a*-OMVs - likely reflects differences in vesicular cargo (gingipains *vs.* leukotoxin/CDT) as well as cell line- and microenvironment-specific responses. Systematic comparative studies using identical cell lines and standardized OMV isolation protocols areneeded to resolve this paradox.

Several important limitations should be noted. First, most mechanistic insights into *F. n*-OMVs are derived from colorectal cancer models; direct extrapolation to oral carcinogenesis requires caution, as the gut and oral microenvironments differ substantially in pH, microbiota composition, immune landscape, and tissue architecture^[70]^. Second, the majority of OSCC studies are *in vitro* or use xenograft mouse models, which may not fully recapitulate the complex tumor microenvironment of human OSCC. Third, clinical correlational data (e.g., higher *P. gingivalis* abundance in OSCC tissues) do not establish causality. Future studies specifically using oral cancer models - such as patient-derived organoids or orthotopic mouse models - are needed to validate whether the same mechanisms operate in the oral niche.

### Alzheimer’s disease

Emerging evidence has implicated periodontal pathogens, particularly *P. gingivalis*, in AD pathogenesis, with OMVs proposed as key mediators of the “oral-brain axis”^[71]^. As detailed in Table 2, O-BEVs from *P. gingivalis* and *A. actinomycetemcomitans* have been reported to contribute to AD pathology through three interconnected mechanisms: (i) blood-brain barrier (BBB) disruption; (ii) neuroinflammation; and (iii) tau hyperphosphorylation, among which *A. a*-OMVs are involved only in BBB disruption and neuroinflammation.

**Table 2:** The role of O-BEVs in systemic and other related diseases

**Diseases**	**O-BEVs source**	**Key effector molecules**	**Core effect**	**Key pathways/targets**	**Ref.**
Alzheimer’s disease	*P. gingivalis* OMVs	Gingipains, LPS	Neuroinflammation, tau hyperphosphorylation	NLRP3/NF-κB; ↑IL-1β/IL-6/TNF-α; tau (Thr231)	[72-77]
	*A. actinomycetemcomitans* OMVs	LPS, RNA	Neuroinflammation	TLR4/MyD88, TLR8/NF-κB; cross BBB	[72,78]
Atherosclerosis	*P. gingivalis* OMVs	Gingipains, LPS, histone H3	Calcification, endothelial dysfunction, foam cells	ERK1/2-RUNX2; ↓eNOS; PECAM-1 disruption	[80-83]
	*T. denticola* OMVs	LPS, MCP-1	Monocyte chemotaxis	↑IL-8/MCP-1 in endothelium	[84]
	*F. nucleatum* OMVs	LPS	Foam cell formation	↑CD36; ↑ox-LDL uptake	[85]
Diabetes mellitus	*P. gingivalis* OMVs	Gingipains	Insulin resistance, retinopathy	↓Akt/GSK-3β; PAR-2 signaling	[87,88]
	*F. nucleatum* OMVs	LPS	Insulin resistance	TLR4-mediated inflammation	[89,90]
RA	*P. gingivalis* OMVs	PPAD, gingipains	Autoimmunity	Protein citrullination → ACPA; ↑TNF-α/IL-1/IL-6	[93-97]
	*F. nucleatum* OMVs	FadA	Synovial inflammation	Rab5a/YB-1 activation	[98]
APOs	*P. gingivalis* OMVs	Gingipains	Trophoblast dysfunction, abortion, offspring neurodevelopmental defects	↓Glucose metabolism; NET dysregulation; ↑p-Tau Thr231	[101-106]
Osteoporosis	*P. gingivalis*, *T. forsythia* OMVs; *F. alocis*, *S. oralis* CMVs	Lipoproteins, LPS	Osteoclast differentiation	TLR2 activation	[109]
	*P. gingivalis* OMVs	Unknown	Impaired osteogenesis	↓CPT2; ↓FAO	[110]
	*F. alocis* CMVs	Unknown	Bone loss	↓Osteogenic markers; ↑CTX-1	[111,112]
Respiratory diseases	*P. gingivalis* OMVs	Histone H3	Lung epithelial injury	NF-κB; ↑pro-inflammatory cytokines	[113,114]
Hepatic steatosis	*F. alocis* CMVs	Unknown	Hepatic steatosis	TLR-2, PAI-1	[115]
OLP	*P. gingivalis*, *A. actinomycetemcomitans* OMVs	LPS, RNA	Inflammation	STAT3; ↑TNF-α/IL-6/IL-8	[116]
HIV infection	*P. gingivalis* OMVs	Unknown	Enhanced viral infectivity	Mucosal transmission vector	[117]

O-BEVs: Oral bacterial extracellular vesicles; *P. gingivalis*: *Porphyromonas gingivalis*; OMVs: outer membrane vesicles; LPS: lipopolysaccharide; NLRP3: NOD-, LRR- and pyrin domain-containing protein 3; NF-κB: nuclear factor kappa B; IL-1β: interleukin-1 beta; IL-6: interleukin-6; TNF-α: tumor necrosis factor alpha; *A. actinomycetemcomitans*: *Aggregatibacter actinomycetemcomitans*; TLR8/4/2: Toll-like receptor 8/4/2; BBB: blood-brain barrier; ERK1/2: extracellular signal-regulated kinase 1/2; RUNX2: Runt-related transcription factor 2; eNOS: endothelial nitric oxide synthase; PECAM-1: platelet endothelial cell adhesion molecule-1; *T. denticola*: *Treponema denticola*; MCP-1: monocyte chemoattractant protein-1; IL-8: interleukin-8; *F. nucleatum*: *Fusobacterium nucleatum*; LDL: low-density lipoprotein; Akt: protein kinase B; GSK-3β: glycogen synthase kinase-3 beta; PAR-2: protease-activated receptor-2; RA: rheumatoid arthritis; PPAD: peptidylarginine deiminase; ACPA: anti-citrullinated protein antibody; IL-1: interleukin-1; FadA: Fusobacterium adhesin A; APOs: adverse pregnancy outcomes; NET: neutrophil extracellular trap; p-Tau: phosphorylated tau; *T. forsythia*: *Tannerella forsythia*; *F. alocis*: *Filifactor alocis*; *S. oralis*: *Streptococcus oralis*; CMVs: cytoplasmic membrane vesicles; CPT2: carnitine palmitoyltransferase 2; FAO: fatty acid oxidation; CTX-1: C-terminal telopeptide of type I collagen; OLP: oral lichen planus; PAI-1: plasminogen activator inhibitor-1; STAT3: signal transducer and activator of transcription 3; HIV: Human Immunodeficiency Virus.

While intact *P. gingivalis* bacteria induce pronounced systemic inflammation, OMVs alone are sufficient to induce BBB disruption and promote neuroinflammation, suggesting a distinct pathogenic mechanism^[72]^. The *P. g*-OMVs activate the NLRP3 inflammasome in microglia, leading to IL-1β release^[73]^. This inflammatory cascade subsequently induces tau hyperphosphorylation at the Thr231 site in neurons and impairs spatial memory and learning in middle-aged mice^[74]^. The LPS and gingipains carried by these OMVs have been implicated as key effector molecules driving these effects. For example, *P. g*-OMVs significantly increase the expression of IL-6, TNF-α, IL-8 and IL-1β in microglial cells, whereas a gingipain-deficient KDP163 strain fails to upregulate these genes^[75]^. Moreover, LPS from *P. g*-OMVs can activate the NF-κB signaling pathway via TLR4, causing substantial release of pro-inflammatory cytokines and further aggravating neuroinflammation^[76]^. In a mouse model, the *P. g*-OMVs significantly increased the expression of IL-1β and TNF-α in the hippocampus and cortex, activated astrocytes and microglia, and resulted in memory dysfunction^[77]^.

In contrast to the gingipain-centered mechanisms of *P. g*-OMVs, the *A. a*-OMVs have been reported to induce neuroinflammation primarily through their RNA cargo. These OMVs can alter action potentials in trigeminal ganglion (TG) neurons^[72]^. *In vitro* and animal studies have suggested that these OMVs may cross the BBB and deliver extracellular RNA into brain monocytes and microglial cells, activating TLR4/MyD88 and TLR8-dependent NF-κB pathways and promoting TNF-α and IL-6 secretion^[78]^. This finding suggests that different O-BEVs may converge on similar neuroinflammatory endpoints through distinct molecular routes - a nuance that would be missed by a simple enumeration of individual mechanisms.

### Atherosclerosis

Epidemiological and mechanistic studies have increasingly linked periodontitis to atherosclerosis, with O-BEVs emerging as potential mediators of this association^[79]^. As detailed in Table 2, O-BEVs from *P. gingivalis*, *F. nucleatum*, and *T. denticola* have been implicated in atherosclerosis through three interconnected mechanisms: (i) endothelial dysfunction and barrier disruption; (ii) vascular calcification and smooth muscle cell dysfunction; and (iii) foam cell formation and lipid accumulation.

Studies have shown that *P. g*-OMVs promote vascular smooth muscle cell (VSMC) calcification in a concentration-dependent manner via the extracellular signal-regulated kinase 1/2-Runt-related transcription factor 2 (ERK1/2-RUNX2) pathway, a hallmark of atherosclerotic progression^[80]^. Using *in vitro* and *in vivo* experiments, Farrugia *et al.* confirmed that *P. g*-OMVs increased vascular permeability by disrupting platelet endothelial cell adhesion molecule-1 (PECAM-1) in endothelial cells in a gingipain-dependent manner and enhanced vascular edema and mortality in a zebrafish model. Further evidence indicates that gingipain-containing OMVs impair intercellular contacts by cleaving junctional proteins such as CD31, thereby facilitating trans-endothelial migration of immune cells into the arterial intima^[81]^. Moreover, *P. g*-OMVs have been reported to suppress the expression of endothelial nitric oxide synthase (eNOS) in human umbilical vein endothelial cells (HUVECs) and mouse aortic endothelium, potentially reducing NO secretion and impairing endothelial function^[82]^. Once monocytes have infiltrated into the arterial wall, *P. g*-OMVs contribute to the aggregation and modification of low-density lipoprotein (LDL), promoting foam cell formation and accelerated lipid accumulation within the vessel wall. Fleetwood *et al.* found that stimulation of macrophages with *P. g*-OMVs triggered a metabolic shift from oxidative phosphorylation to glycolysis, accompanied by secretion of numerous inflammatory mediators. This metabolic reprogramming may activate the inflammasome and induces pyroptosis, resulting in the release of inflammatory cytokines and cytoplasmic components into the extracellular environment, thereby sustaining local inflammation and amplifying pyroptotic cell death^[83]^.

Beyond *P. gingivalis*, *T. d*-OMVs can activate endothelial cells by inducing the expression of IL-8 and MCP-1, which may facilitate chemotaxis and aggregation of monocytes - an early step in atherogenesis^[84]^. Additionally, the *F. n*-OMVs have been reported to upregulate the scavenger receptor CD36 via the TLR4/NF-κB pathway, enhancing the uptake of oxidized LDL (ox-LDL) and accelerating foam cell formation^[85]^.

In summary, OMVs derived from key periodontal pathogens do not act in isolation. Instead, they likely form a complex pathogenic network that collectively accelerates the initiation and progression of atherosclerosis through multiple interconnected mechanisms, including disruption of endothelial homeostasis, dysregulation of lipid metabolism, and perpetuation of chronic inflammation.

### Diabetes mellitus

Epidemiological and mechanistic studies have long suggested a bidirectional relationship between periodontitis and diabetes mellitus (DM): periodontitis can impair glycemic control, insulin action, and diabetic outcomes, while DM can heighten periodontitis severity through delayed healing and enhanced infection risk^[86]^. As detailed in Table 2, *P. g*-OMVs and *F. n*-OMVs have been implicated in the pathogenesis and complications of diabetes through three interconnected mechanisms: (i) direct impairment of hepatic insulin signaling; (ii) exacerbation of diabetic complications (e.g., retinopathy); and (iii) promotion of systemic and intestinal inflammation that may worsen insulin resistance.

The *P. g*-OMVs can deliver active gingipains to the liver. In HepG2 hepatocytes, these vesicles attenuate insulin-induced protein kinase B (Akt)/glycogen synthase kinase-3 beta (GSK-3β) signaling in a gingipain-dependent manner, thereby disrupting hepatic glucose metabolism and potentially promoting the onset and progression of DM^[87]^. Furthermore, in mice, *P. g*-OMVs worsen diabetic retinopathy (DR), an effect that may involve mitochondrial-associated cell death and endothelial dysfunction triggered by PAR-2 signaling in human retinal microvascular endothelial cells^[88]^. Emerging evidence also indicates that periodontal pathogens and their vesicles may promote insulin resistance in peripheral tissues. For instance, OMVs derived from other periodontopathic bacteria such as *F. nucleatum* have been implicated in impairing insulin signaling in adipocytes through TLR4-mediated inflammatory pathways^[89]^. According to Engevik *et al.*, *F. n*-OMVs activate TLR4 on intestinal epithelial cells. This may trigger a signaling cascade involving ERK, CREB, and NF-κB, which then stimulates the release of pro-inflammatory cytokines (e.g., IL-8, TNF-α) and promotes intestinal inflammation^[89]^. Moreover, systemic inflammation triggered by OMVs-induced release of pro-inflammatory cytokines may further aggravate pancreatic β-cell dysfunction and insulin resistance^[90]^. These mechanisms collectively underscore the role of O-BEVs as potential contributors to the pathogenesis and complications of diabetes, suggesting the potential benefits of oral microbiome management in glycemic control.

### RA

Autoantibodies produced in RA patients, such as rheumatoid factor (RF) and ACPAs, bind synovial antigens, activate the complement and trigger joint inflammation^[91]^. Epidemiological and mechanistic studies have long suggested an association between periodontitis and RA, with O-BEVs emerging as potential mediators linking oral dysbiosis to joint autoimmunity^[92]^. As detailed in Table 2, *P. g*-OMVs and *F. n*-OMVs have been linked to RA pathogenesis through two distinct but potentially complementary mechanisms: (i) PPAD-mediated protein citrullination and ACPA generation; and (ii) FadA-mediated activation of synovial macrophages.

PPAD in *P. g*-OMVs serves as a key effector molecule mediating the pathogenic role of these vesicles in RA. The PPAD catalyzes the citrullination of host proteins such as fibrinogen and vimentin, generating ACPAs that are highly specific to RA and triggering inflammatory responses in the synovium^[93]^. Notably, other virulence factors within *P. g*-OMVs may act synergistically with PPAD. For instance, gingipains cleave proteins at arginine residues, whereas PPAD preferentially citrullinates C-terminal arginine residues on polypeptide chains^[94]^. Furthermore, elevated levels of TNF-α, IL-1β, and IL-6 in both serum and synovial fluid promote inflammatory responses and exacerbate joint damage^[95]^. The O-BEVs derived DNA has been detected not only in serum^[96]^, but also in synovial fluid^[97]^, providing additional evidence for the systemic dissemination of oral bacterial components to the joint microenvironment.

A positive correlation exists between *F. nucleatum* abundance in RA patients and disease severity. *F. n*-OMVs carrying FadA can reach the joints and provoke local inflammation, and FadA targets synovial macrophages, resulting in activation of Rab5a GTPase (a vesicular trafficking regulator) and YB-1 (an inflammatory pathway modulator)^[98]^. Moreover, certain O-BEVs-associated molecules can act as damage-associated molecular patterns (DAMPs), sustaining chronic activation of innate immune pathways within the joint microenvironment. Collectively, these mechanisms underscore how O-BEVs orchestrate a breach in immune tolerance and fuel autoimmunity in RA, providing a compelling mechanistic basis for the established association between periodontitis and RA.

### Adverse pregnancy outcomes

Accumulating epidemiological data have revealed that adverse pregnancy outcomes (APOs) are strongly associated with periodontitis^[99,100]^. As detailed in Table 2, *P. g-*OMVs have been implicated in APOs through three interconnected mechanisms: (i) direct targeting and dysfunction of trophoblast cells at the maternal-fetal interface; (ii) indirect damage mediated by host immune cell-derived EVs; and (iii) offspring transgenerational neurodevelopmental alterations.

The *P. g*-OMVs can directly target the maternal-fetal interface and disrupt normal pregnancy progression. For example, when pregnant mice were intraperitoneally injected with *P. g*-OMVs, fluorescent signals were subsequently detected at embryo implantation sites^[101]^. Low doses of *P. g*-OMVs reduced fetal and placental weights, whereas high doses resulted in fetal loass^[102]^. *In vitro* experiments from the same study revealed that *P. g*-OMVs decreased glucose uptake and glycolysis in trophoblast cells, impairing their migration and invasion capabilities. Proper migration of trophoblasts and endothelial cells is crucial for remodeling spiral arteries at the maternal-fetal interface^[101,102]^. Under physiological conditions, trophoblasts contribute to placental homeostasis by inactivating neutrophils through the suppression of ROS release and neutrophil extracellular trap (NET) formation^[103]^. Conditioned medium from *P. g*-OMV-treated trophoblast cells, however, promoted neutrophil chemotaxis and increased the production of ROS, IL-8, and TNF-α, potentially disrupting placental homeostasis and thereby inducing APOs^[104]^.

Beyond the direct effects of bacterial OMVs, the pathogenic impact of oral bacteria can be further amplified by host immune cells. EVs derived from *P. gingivalis*-infected macrophages (*P. g*-inf EVs) translocated to the fetoplacental unit and impaired fetal development, as evidenced by reduced fetal size and weight. Histological analysis of the placenta in the *P. g*-inf EV-injected group revealed disorganized vasculature, impaired angiogenesis, and compromised placental function, and proteomic analysis indicated a significant downregulation of VEGFR1 expression in the experimental placentas^[105]^.

More critically, maternal exposure to *P. g*-OMVs during pregnancy may exert intergenerational effects, directly disrupting the neurodevelopmental programming of offspring. Further evidence demonstrates that maternal exposure to *P. g*-OMVs induces marked neurodevelopmental alterations in the offspring. These include the suppression of key molecules (IL-6, Cux1 and SatB2) implicated in cortical development and neuronal differentiation, concurrent with an elevation in the Alzheimer’s disease-associated phospho-Tau (Thr^231^) and changes in embryonic cortical neuron density^[106]^. The ability of O-BEVs to translocate systemically and disrupt key physiological processes during pregnancy underscores their potential role as mediators of the “oral-systemic axis” in reproductive pathology. Further research is warranted to explore the translational potential of targeting O-BEVs or their cargo for the prevention and management of periodontitis-associated APOs.

In summary, O-BEVs establish a multiple pathogenic axis from local infection to systemic reproductive pathology and even intergenerational health effects, spanning from direct damage to placental trophoblasts, to secondary immune-mediated damage, and finally to interference with offspring neurodevelopment. Future research is essential to thoroughly explore the translational potential of targeting these vesicles or their pathogenic components, thereby paving the way for novel intervention strategies to prevent and manage periodontitis-associated APOs.

### Osteoporosis

Osteoporosis and periodontitis exhibit a bidirectional relationship^[107]^. Osteoporotic individuals face a twofold greater risk of developing periodontitis than healthy people^[108]^. In both conditions, bone loss localized to alveolar bone in periodontitis or systemic in osteoporosis - is tied to heightened osteoclast differentiation and reduced osteogenesis. As detailed in Table 2, O-BEVs from multiple oral pathogens contribute to bone loss through two principal mechanisms: (i) promotion of osteoclast differentiation and (ii) suppressed osteogenesis.

Lipoproteins or LPS from O-BEVs from *F. alocis*, *P. gingivalis*, *T. forsythia*, and *S. oralis* drive osteoclast differentiation through activation of TLR2^[109]^. Emerging evidence indicates that *P. g*-OMVs exacerbate osteoporosis by impairing mitochondrial dynamics, which downregulates the protein level of CPT2. This suppression of fatty acid oxidation (FAO) subsequently compromises ATP production in osteoblasts, ultimately contributing to bone loss^[110]^. Furthermore, when added to osteogenic medium, *F. a*-CMVs suppressed bone formation in a dose-dependent manner, as indicated by reduced expression of osteogenic marker genes^[111]^. Following intraperitoneal injection of DiO-labeled *F. a*-CMVs into mice, strong fluorescence was detected in the tibiae and femora, accompanied by decreased trabecular bone volume and elevated levels of the bone resorption marker CTX-1^[112]^. These findings suggest that O-BEVs can travel to long bones and trigger bone loss, potentially via TLR2 signaling.

### Other diseases

Beyond the systemic conditions discussed above, O-BEVs have also been implicated in several other human diseases, including respiratory diseases, hepatic steatosis, oral lichen planus (OLP), and HIV-1 infection, as summarized in Table 2.

Respiratory diseases: The *P. g*-OMVs trigger cell death in lung epithelial cells by disrupting the epithelial barrier, suggesting that these vesicles may be an important factor linking periodontitis to respiratory diseases^[113]^. Notably, *P. g*-OMVs are enriched in core histones (e.g., H3) and translocate to the lungs, liver, and kidneys of mice. Both *P. g*-OMVs and recombinant H3 activated the NF-κB pathway, contributing to increased levels of pro-inflammatory cytokines in human lung epithelial A549 cells. The *P. g*-inf EVs induced lung injury, including edema, vascular congestion, inflammation, and collagen deposition, which was associated with alveolar damage^[114]^.

Hepatic steatosis: The *F. a-*CMVs have been associated with hepatic steatosis, particularly through increasing this condition in mice on a low-fat diet through mechanisms involving TLR-2 and PAI-1^[115]^.

OLP: *A. a*-OMVs and *P. g*-OMVs did not affect cell viability but potently increased the mRNA levels of TNF-α, IL-6, and IL-8. Moreover, these vesicles activated the STAT3 signaling pathway, as evidenced by increased phosphorylation and simultaneous upregulation of IL-1β mRNA, along with promoted NLRP3 protein accumulation. These changes suggest that the inflammasome complex may be activated^[116]^.

HIV-1 infection: In MT4 cells, *P. g*-OMVs promoted HIV-1 infection even when viral loads alone were too low to establish a productive infection. This suggests that OMVs may act as vectors for mucosal HIV transmission, thereby facilitating infection establishment and enhancing viral infectivity^[117]^.

## CONCLUSION

O-BEVs function as essential communicators and pathogenicity vectors in the oral-systemic axis. Their cargo - which includes virulence proteins (e.g., gingipains, PPAD, FadA), immunomodulatory lipids (e.g., specialized LPS, cardiolipin), and regulatory nucleic acids (e.g., sRNA, eRNA) - enables them to disrupt local homeostasis and propagate systemic inflammation^[32,34]^. Specifically, O-BEVs from periodontopathic bacteria such as *P. gingivalis*, *T. forsythia*, and *F. nucleatum* drive periodontal tissue destruction via TLR/NF-κB and NLRP3 inflammasome activation^[52,55]^, while *S. mutans* extracellular vesicles promote cariogenic biofilm formation through GTF-mediated EPS synthesis^[58]^.

Beyond the oral cavity, O-BEVs disseminate and contribute to systemic pathologies through distinct mechanisms: In Alzheimer’s disease, OMVs compromise the BBB and induce neuroinflammation and tau hyperphosphorylation^[76,77]^. In atherosclerosis, they promote vascular calcification and endothelial dysfunction^[81,84]^. Their role in RA is highlighted by PPAD-mediated protein citrullination and ACPA generation^[33,94,98]^, while in diabetes, gingipain delivery impairs hepatic insulin signaling^[88]^. Moreover, O-BEVs influence conditions ranging from APOs (via trophoblast dysfunction)^[102]^ to osteoporosis (through enhanced osteoclastogenesis)^[110,111]^.

It is important to recognize that O-BEV research remains an emerging field. Most mechanistic insights derive from preclinical studies, and direct evidence of clinical relevance is limited. Heterogeneity in isolation methods, lack of standardized quantification, and the predominance of studies on *P. gingivalis* constrain the generalizability of current findings. Nevertheless, the conceptual framework of the “oral-systemic axis” mediated by O-BEVs offers promising opportunities for future diagnostics and therapeutics.

This synthesis underscores that O-BEVs are not merely bystanders but active mechanistic connectors between oral dysbiosis and systemic diseases. Future translational efforts should focus on three O-BEV-centric directions: First, developing salivary O-BEV-based biomarkers for early, non-invasive detection of periodontitis and associated systemic diseases by leveraging the stable nucleic acid and protein cargo of O-BEVs^[118,119]^. Their RNA content (e.g., inflammation-related miRNAs upregulated upon *P. gingivalis* infection) has shown promise as diagnostic biomarkers^[119]^. Second, designing specific inhibitors against O-BEV-borne virulence factors as an anti-virulence strategy that spares the commensal microbiota. The clinical-stage lysine-gingipain inhibitor LHP588, an orally available, brain-penetrant compound for *P. gingivalis*-associated Alzheimer’s disease, has entered a Phase 2 trial (SPRING Trial) supported by a $49.2 million NIA grant, offering proof of concept for targeting O-BEVs virulence^[120]^. Third, engineering non-pathogenic or probiotic O-BEV as therapeutic delivery vehicles. Probiotic *Escherichia coli* Nissle 1917-derived OMVs have been engineered to load platinum nanoparticles and curcumin (Cur@OMV-Pt nanocomposite), which integrate antibacterial, anti-inflammatory, and tissue-healing properties for treating bacterially infected oral ulcers^[121]^. Advancing these O-BEV-focused avenues will be critical for realizing precision medicine along the “oral-systemic axis”.

## DECLARATIONS

### AI and AI-assisted tools statement

During the preparation of this manuscript, the AI tool Deepseek-V3.2 was used solely for language editing and grammar checking. The tool did not influence the study design, data collection, analysis, interpretation, or the scientific content of the work. All authors take full responsibility for the accuracy, integrity, and final content of the manuscript.

### Copyright

© The Author(s) 2026.

## References

[1] Dame-Teixeira N, Do T, Deng D (2025). The oral microbiome and us. Adv Exp Med Biol.

[2] Baker JL, Mark Welch JL, Kauffman KM, McLean JS, He X (2024). The oral microbiome: diversity, biogeography and human health. Nat Rev Microbiol.

[3] Arweiler NB, Netuschil L (2016). The oral microbiota. Adv Exp Med Biol.

[4] Wade WG (2013). The oral microbiome in health and disease. Pharmacol Res.

[5] Tuganbaev T, Yoshida K, Honda K (2022). The effects of oral microbiota on health. Science.

[6] Kleinstein SE, Nelson KE, Freire M (2020). Inflammatory networks linking oral microbiome with systemic health and disease. J Dent Res.

[7] Peng X, Cheng L, You Y (2022). Oral microbiota in human systematic diseases. Int J Oral Sci.

[8] Bosshardt DD (2018). The periodontal pocket: pathogenesis, histopathology and consequences. Periodontol 2000.

[9] Kunath BJ, De Rudder C, Laczny CC, Letellier E, Wilmes P (2024). The oral-gut microbiome axis in health and disease. Nat Rev Microbiol.

[10] Hernández-Cabanyero C, Vonaesch P (2024). Ectopic colonization by oral bacteria as an emerging theme in health and disease. FEMS Microbiol Rev.

[11] Asikainen S, Alaluusua S (1993). Bacteriology of dental infections. Eur Heart J.

[12] Xie J, Li Q, Haesebrouck F, Van Hoecke L, Vandenbroucke RE (2022). The tremendous biomedical potential of bacterial extracellular vesicles. Trends Biotechnol.

[13] Ho MY, Liu S, Xing B (2024). Bacteria extracellular vesicle as nanopharmaceuticals for versatile biomedical potential. Nano Converg.

[14] Moghaddam ZS, Dehghan A, Halimi S (2025). Bacterial extracellular vesicles: bridging pathogen biology and therapeutic innovation. Acta Biomater.

[15] Toyofuku M, Schild S, Kaparakis-Liaskos M, Eberl L (2023). Composition and functions of bacterial membrane vesicles. Nat Rev Microbiol.

[16] Effah CY, Ding X, Drokow EK, Li X, Tong R, Sun T (2024). Bacteria-derived extracellular vesicles: endogenous roles, therapeutic potentials and their biomimetics for the treatment and prevention of sepsis. Front Immunol.

[17] Yates AG, Pink RC, Erdbrügger U (2022). In sickness and in health: the functional role of extracellular vesicles in physiology and pathology in vivo: Part I: Health and Normal Physiology: Part I: Health and Normal Physiology. J Extracell Vesicles.

[18] Yates AG, Pink RC, Erdbrügger U (2022). In sickness and in health: the functional role of extracellular vesicles in physiology and pathology in vivo: Part II: Pathology: Part II: Pathology. J Extracell Vesicles.

[19] Kaparakis-Liaskos M, Ferrero RL (2015). Immune modulation by bacterial outer membrane vesicles. Nat Rev Immunol.

[20] Jeong GJ, Khan F, Tabassum N, Cho KJ, Kim YM (2024). Bacterial extracellular vesicles: modulation of biofilm and virulence properties. Acta Biomater.

[21] Xia X, Fang Y, Zhong J, Li F, Jiang L (2025). Biomimetic strategies of cell membrane vesicles driven by pathogen-host interactions: novel insights into antimicrobial immunotherapy and infection prevention. Front Immunol.

[22] Deng DK, Zhang JJ, Gan D (2022). Roles of extracellular vesicles in periodontal homeostasis and their therapeutic potential. J Nanobiotechnology.

[23] Schwechheimer C, Kuehn MJ (2015). Outer-membrane vesicles from Gram-negative bacteria: biogenesis and functions. Nat Rev Microbiol.

[24] Costa TRD, Felisberto-Rodrigues C, Meir A (2015). Secretion systems in Gram-negative bacteria: structural and mechanistic insights. Nat Rev Microbiol.

[25] Briaud P, Carroll RK (2020). Extracellular vesicle biogenesis and functions in Gram-positive bacteria. Infect Immun.

[26] (2024). Welsh JA, Goberdhan DCI, O’Driscoll L, et al.; MISEV Consortium. Minimal information for studies of extracellular vesicles (MISEV2023): from basic to advanced approaches. J Extracell Vesicles.

[27] Zhang H, Lin Y, Li S (2024). Effects of bacterial extracellular vesicles derived from oral and gastrointestinal pathogens on systemic diseases. Microbiol Res.

[28] (2021). Ñahui Palomino RA, Vanpouille C, Costantini PE, Margolis L. Microbiota-host communications: bacterial extracellular vesicles as a common language. PLoS Pathog.

[29] Gan Y, Zhao G, Wang Z, Zhang X, Wu MX, Lu M (2023). Bacterial membrane vesicles: physiological roles, infection immunology, and applications. Adv Sci.

[30] Toyofuku M, Nomura N, Eberl L (2019). Types and origins of bacterial membrane vesicles. Nat Rev Microbiol.

[31] Furuyama N, Sircili MP (2021). Outer membrane vesicles (OMVs) produced by Gram-negative bacteria: structure, functions, biogenesis, and vaccine application. Biomed Res Int.

[32] Zhang Z, Liu D, Liu S, Zhang S, Pan Y (2020). The role of *Porphyromonas gingivalis* outer membrane vesicles in periodontal disease and related systemic diseases. Front Cell Infect Microbiol.

[33] Ahmadi P, Mahmoudi M, Kheder RK (2023). Impacts of *Porphyromonas gingivalis* periodontitis on rheumatoid arthritis autoimmunity. Int Immunopharmacol.

[34] Zhang L, Leng XX, Qi J (2024). The adhesin RadD enhances *Fusobacterium nucleatum* tumour colonization and colorectal carcinogenesis. Nat Microbiol.

[35] Zhang L, Zhang D, Liu C (2024). Outer membrane vesicles derived from *Fusobacterium nucleatum* trigger periodontitis through host overimmunity. Adv Sci.

[36] Kato S, Kowashi Y, Demuth DR (2002). Outer membrane-like vesicles secreted by *Actinobacillus actinomycetemcomitans* are enriched in leukotoxin. Microb Pathog.

[37] Rompikuntal PK, Thay B, Khan MK (2012). Perinuclear localization of internalized outer membrane vesicles carrying active cytolethal distending toxin from *Aggregatibacter actinomycetemcomitans*. Infect Immun.

[38] Rosen G, Naor R, Rahamim E, Yishai R, Sela MN (1995). Proteases of *Treponema denticola* outer sheath and extracellular vesicles. Infect Immun.

[39] Abiko Y, Nagano K, Yoshida Y, Yoshimura F (2014). Characterization of *Treponema denticola* mutants defective in the major antigenic proteins, Msp and TmpC. PLoS One.

[40] Lim Y, Kim HY, Han D, Choi BK (2023). Proteome and immune responses of extracellular vesicles derived from macrophages infected with the periodontal pathogen *Tannerella forsythia*. J Extracell Vesicles.

[41] Kim HY, Lim Y, An SJ, Choi BK (2020). Characterization and immunostimulatory activity of extracellular vesicles from *Filifactor alocis*. Mol Oral Microbiol.

[42] Nakamura T, Iwabuchi Y, Hirayama S (2020). Roles of membrane vesicles from *Streptococcus mutans* for the induction of antibodies to glucosyltransferase in mucosal immunity. Microb Pathog.

[43] Peri F, Granucci F, Weiss J (2015). “Endotoxin, TLR4 signaling and beyond”. Mol Immunol.

[44] Roier S, Zingl FG, Cakar F (2016). A novel mechanism for the biogenesis of outer membrane vesicles in Gram-negative bacteria. Nat Commun.

[45] Gui MJ, Dashper SG, Slakeski N, Chen YY, Reynolds EC (2016). Spheres of influence: *Porphyromonas gingivalis* outer membrane vesicles. Mol Oral Microbiol.

[46] Cecil JD, O’Brien-Simpson NM, Lenzo JC (2016). Differential responses of pattern recognition receptors to outer membrane vesicles of three periodontal pathogens. PLoS One.

[47] Wang J, Liu C, Cutler J, Ivanovski S, Lee RS, Han P (2024). Microbial- and host immune cell-derived extracellular vesicles in the pathogenesis and therapy of periodontitis: a narrative review. J Periodontal Res.

[48] Choi JW, Kim SC, Hong SH, Lee HJ (2017). Secretable small RNAs via outer membrane vesicles in periodontal pathogens. J Dent Res.

[49] Villanueva-Carmona T, Cedó L, Madeira A (2023). SUCNR1 signaling in adipocytes controls energy metabolism by modulating circadian clock and leptin expression. Cell Metab.

[50] Luo R, Chang Y, Liang H (2023). Interactions between extracellular vesicles and microbiome in human diseases: new therapeutic opportunities. Imeta.

[51] Jing L, Wang HY, Zhang N (2025). Critical roles of extracellular vesicles in periodontal disease and regeneration. Stem Cells Transl Med.

[52] Mahendrarajan V, Lazarus HPS, Muthukaliannan GK, Varghese S, Easwaran N (2025). Membrane vesicles from Red Complex bacteria: key players in oral pathogenesis, immune disruption, systemic diseases, and therapeutic insights. Front Oral Health.

[53] Uemura Y, Hiroshima Y, Tada A (2022). *Porphyromonas gingivalis* outer membrane vesicles stimulate gingival epithelial cells to induce pro-inflammatory cytokines via the MAPK and STING pathways. Biomedicines.

[54] Friedrich V, Gruber C, Nimeth I (2015). Outer membrane vesicles of *Tannerella forsythia*: biogenesis, composition, and virulence. Mol Oral Microbiol.

[55] Zhao Y, Chen J, Tian Y, Huang H, Zhao F, Deng X (2025). *Treponema denticola* major surface protein (Msp): a key player in periodontal pathogenicity and immune evasion. Arch Microbiol.

[56] Chen G, Sun Q, Cai Q, Zhou H (2022). Outer membrane vesicles from *Fusobacterium nucleatum* switch M0-like macrophages toward the M1 phenotype to destroy periodontal tissues in mice. Front Microbiol.

[57] Zhang R, Li G, Wu Y, Wang X, Luan Q (2024). Pathogenic mechanisms and potential applications of extracellular vesicles from periodontal pathogens in periodontitis. Front Immunol.

[58] Song G, Li M, Zhou B, Qi H, Guo J (2024). *Streptococcus mutans* outer membrane vesicles affect inflammasome activation and the glycolysis of macrophages. Microb Pathog.

[59] Rainey K, Michalek SM, Wen ZT, Wu H (2019). Glycosyltransferase-mediated biofilm matrix dynamics and virulence of *Streptococcus mutans*. Appl Environ Microbiol.

[60] Cui G, Li P, Wu R, Lin H (2022). *Streptococcus mutans* membrane vesicles inhibit the biofilm formation of *Streptococcus gordonii* and *Streptococcus sanguinis*. AMB Express.

[61] Wu R, Tao Y, Cao Y, Zhou Y, Lin H (2020). *Streptococcus mutans* membrane vesicles harboring glucosyltransferases augment *Candida albicans* biofilm development. Front Microbiol.

[62] Johnson DE, Burtness B, Leemans CR, Lui VWY, Bauman JE, Grandis JR (2020). Head and neck squamous cell carcinoma. Nat Rev Dis Primers.

[63] Liu D, Liu S, Liu J, Miao L, Zhang S, Pan Y (2021). sRNA23392 packaged by *Porphyromonas gingivalis* outer membrane vesicles promotes oral squamous cell carcinomas migration and invasion by targeting desmocollin-2. Mol Oral Microbiol.

[64] Zeng Y, Wang Y, Shi X (2025). *Porphyromonas gingivalis* outer membrane vesicles augments proliferation and metastasis of oral squamous cell carcinoma cells. BMC Oral Health.

[65] Chen Q, Pang X, Liu K (2025). *Porphyromonas gingivalis* outer membrane vesicles promote oral tumorigenesis through suppressing innate immune surveillance. Microbiol Res.

[66] Yan L, Wu F, Jiao J (2025). *Stomatobaculum longum*-derived extracellular vesicles enhance oral squamous cell carcinoma malignancy through BRCA1/EXO1/TP53BP1 modulation. Int J Nanomedicine.

[67] Chen G, Gao C, Jiang S (2024). *Fusobacterium nucleatum* outer membrane vesicles activate autophagy to promote oral cancer metastasis. J Adv Res.

[68] Metsäniitty M, Hasnat S, Öhman C (2024). Extracellular vesicles from *Aggregatibacter actinomycetemcomitans* exhibit potential antitumorigenic effects in oral cancer: a comparative* in vitro* study. Arch Microbiol.

[69] Ha JY, Choi SY, Kim SJ, Seog KJ, Hong SH, Lee HJ (2025). Transcriptome analysis of HNSCC by *Aggregatibacter actinomycetemcomitans* extracellular vesicles. Oral Dis.

[70] Suárez LJ, Arboleda S, Angelov N, Arce RM (2021). Oral versus gastrointestinal mucosal immune niches in homeostasis and allostasis. Front Immunol.

[71] Liu S, Butler CA, Ayton S, Reynolds EC, Dashper SG (2024). *Porphyromonas gingivalis* and the pathogenesis of Alzheimer’s disease. Crit Rev Microbiol.

[72] Qiu Y, Zhao Y, He G, Yang D (2025). *Porphyromonas gingivalis* and its outer membrane vesicles induce neuroinflammation in mice through distinct mechanisms. Immun Inflamm Dis.

[73] Dominy SS, Lynch C, Ermini F (2019). *Porphyromonas gingivalis* in Alzheimer’s disease brains: evidence for disease causation and treatment with small-molecule inhibitors. Sci Adv.

[74] Gong T, Chen Q, Mao H (2022). Outer membrane vesicles of *Porphyromonas gingivalis* trigger NLRP3 inflammasome and induce neuroinflammation, tau phosphorylation, and memory dysfunction in mice. Front Cell Infect Microbiol.

[75] Yoshida K, Yoshida K, Seyama M (2023). *Porphyromonas gingivalis* outer membrane vesicles in cerebral ventricles activate microglia in mice. Oral Dis.

[76] Inoue E, Minatozaki S, Shimizu S (2024). Human β-defensin 3 inhibition of *P. gingivalis* LPS-induced IL-1β production by BV-2 microglia through suppression of cathepsins B and L. Cells.

[77] Pritchard AB, Fabian Z, Lawrence CL, Morton G, Crean S, Alder JE (2022). An investigation into the effects of outer membrane vesicles and lipopolysaccharide of *Porphyromonas gingivalis* on blood-brain barrier integrity, permeability, and disruption of scaffolding proteins in a human* in vitro* model. J Alzheimers Dis.

[78] Ha JY, Choi SY, Lee JH, Hong SH, Lee HJ (2020). Delivery of periodontopathogenic extracellular vesicles to brain monocytes and microglial IL-6 promotion by RNA cargo. Front Mol Biosci.

[79] Akhi R, Lavrinienko A, Hakula M (2025). Oral microbiota linking humoral response, periodontitis and atherosclerosis. J Clin Periodontol.

[80] Yang WW, Guo B, Jia WY, Jia Y (2016). *Porphyromonas gingivalis*-derived outer membrane vesicles promote calcification of vascular smooth muscle cells through ERK1/2-RUNX2. FEBS Open Bio.

[81] Farrugia C, Stafford GP, Murdoch C (2020). *Porphyromonas gingivalis* outer membrane vesicles increase vascular permeability. J Dent Res.

[82] Jia Y, Guo B, Yang W, Zhao Q, Jia W, Wu Y (2015). Rho kinase mediates *Porphyromonas gingivalis* outer membrane vesicle-induced suppression of endothelial nitric oxide synthase through ERK1/2 and p38 MAPK. Arch Oral Biol.

[83] Fleetwood AJ, Lee MKS, Singleton W (2017). Metabolic remodeling, inflammasome activation, and pyroptosis in macrophages stimulated by *Porphyromonas gingivalis* and its outer membrane vesicles. Front Cell Infect Microbiol.

[84] Huang X, Xie M, Lu X (2023). The roles of periodontal bacteria in atherosclerosis. Int J Mol Sci.

[85] Kuramitsu HK, Qi M, Kang IC, Chen W (2001). Role for periodontal bacteria in cardiovascular diseases. Ann Periodontol.

[86] Shinjo T, Nishimura F (2024). The bidirectional association between diabetes and periodontitis, from basic to clinical. Jpn Dent Sci Rev.

[87] Seyama M, Yoshida K, Yoshida K (2020). Outer membrane vesicles of *Porphyromonas gingivalis* attenuate insulin sensitivity by delivering gingipains to the liver. Biochim Biophys Acta Mol Basis Dis.

[88] Huang S, Cao G, Dai D (2023). *Porphyromonas gingivalis* outer membrane vesicles exacerbate retinal microvascular endothelial cell dysfunction in diabetic retinopathy. Front Microbiol.

[89] Engevik MA, Danhof HA, Ruan W (2021). *Fusobacterium nucleatum* secretes outer membrane vesicles and promotes intestinal inflammation. mBio.

[90] Berbudi A, Khairani S, Tjahjadi AI (2025). Interplay between insulin resistance and immune dysregulation in type 2 diabetes mellitus: implications for therapeutic interventions. Immunotargets Ther.

[91] Sokolova MV, Schett G, Steffen U (2022). Autoantibodies in rheumatoid arthritis: historical background and novel findings. Clin Rev Allergy Immunol.

[92] Rak D, Kulloli AM, Shetty SK (2024). Correlation between rheumatoid arthritis and chronic periodontitis: a systematic review and meta-analysis. Minerva Dent Oral Sci.

[93] Montgomery AB, Kopec J, Shrestha L (2016). Crystal structure of *Porphyromonas gingivalis* peptidylarginine deiminase: implications for autoimmunity in rheumatoid arthritis. Ann Rheum Dis.

[94] Gabarrini G, Palma Medina LM, Stobernack T (2018). There’s no place like OM: vesicular sorting and secretion of the peptidylarginine deiminase of *Porphyromonas gingivalis*. Virulence.

[95] Yokota K, Sato K, Miyazaki T (2021). Characterization and function of tumor necrosis factor and interleukin-6-induced osteoclasts in rheumatoid arthritis. Arthritis Rheumatol.

[96] (2012). de Smit M, Westra J, Vissink A, Doornbos-van der Meer B, Brouwer E, van Winkelhoff AJ. Periodontitis in established rheumatoid arthritis patients: a cross-sectional clinical, microbiological and serological study. Arthritis Res Ther.

[97] Reichert S, Haffner M, Keyßer G (2013). Detection of oral bacterial DNA in synovial fluid. J Clin Periodontol.

[98] Hong M, Li Z, Liu H (2023). *Fusobacterium nucleatum* aggravates rheumatoid arthritis through FadA-containing outer membrane vesicles. Cell Host Microbe.

[99] Bobetsis YA, Graziani F, Gürsoy M, Madianos PN (2020). Periodontal disease and adverse pregnancy outcomes. Periodontol 2000.

[100] Butera A, Maiorani C, Morandini A (2023). Periodontitis in pregnant women: a possible link to adverse pregnancy outcomes. Healthcare.

[101] Tanai A, Okamura H (2021). The role of extracellular vesicles throughout normal pregnancy and in relation to oral bacteria. J Oral Biosci.

[102] Lara B, Loureiro I, Gliosca L (2023). *Porphyromonas gingivalis* outer membrane vesicles shape trophoblast cell metabolism impairing functions associated to adverse pregnancy outcome. J Cell Physiol.

[103] Aplin JD, Myers JE, Timms K, Westwood M (2020). Tracking placental development in health and disease. Nat Rev Endocrinol.

[104] Lara B, Sassot M, Calo G (2023). Extracellular vesicles of *Porphyromonas gingivalis* disrupt trophoblast cell interaction with vascular and immune cells in an *in vitro* model of early placentation. Life.

[105] Tanai A, Fukuhara Y, Eguchi T (2025). *P. gingivalis*-infected macrophage extracellular vesicles cause adverse pregnancy outcomes. J Dent Res.

[106] Bradley AJ, Mashburn-Warren L, Blalock LC, Scarpetti F, Lauber CL (2025). *Porphyromonas gingivalis* outer membrane vesicles alter cortical neurons and Tau phosphorylation in the embryonic mouse brain. PLoS One.

[107] Yu B, Wang CY (2022). Osteoporosis and periodontal diseases - an update on their association and mechanistic links. Periodontol 2000.

[108] Manjunath SH, Rakhewar P, Nahar P, Tambe V, Gabhane M (2019). Evaluation of the prevalence and severity of periodontal diseases between osteoporotic and nonosteoporotic subjects: a cross-sectional comparative study. J Contemp Dent Pract.

[109] Kim HY, Song MK, Lim Y (2022). Effects of extracellular vesicles derived from oral bacteria on osteoclast differentiation and activation. Sci Rep.

[110] Qiu Q, Yan X, Zhang Z (2025). *Porphyromonas gingivalis* extracellular vesicles exacerbated osteoporosis by disrupting osteoblast mitochondrial dynamics and inhibiting Cpt2-regulated fatty acid oxidation. J Nanobiotechnology.

[111] Song MK, Kim HY, Choi BK, Kim HH (2020). *Filifactor alocis*-derived extracellular vesicles inhibit osteogenesis through TLR2 signaling. Mol Oral Microbiol.

[112] Kim HY, Song MK, Gho YS, Kim HH, Choi BK (2021). Extracellular vesicles derived from the periodontal pathogen *Filifactor alocis* induce systemic bone loss through Toll-like receptor 2. J Extracell Vesicles.

[113] He Y, Shiotsu N, Uchida-Fukuhara Y (2020). Outer membrane vesicles derived from *Porphyromonas gingivalis* induced cell death with disruption of tight junctions in human lung epithelial cells. Arch Oral Biol.

[114] Yoshida K, Yoshida K, Fujiwara N (2021). Extracellular vesicles of *P. gingivalis*-infected macrophages induce lung injury. Biochim Biophys Acta Mol Basis Dis.

[115] Kim HY, Lim Y, Jang JS (2024). Extracellular vesicles from periodontal pathogens regulate hepatic steatosis via Toll-like receptor 2 and plasminogen activator inhibitor-1. J Extracell Vesicles.

[116] Kim YG, Song HJ, Kim HJ (2025). Bacterial extracellular vesicles as potential promoting factors for oral lichen planus pathogenesis. Inflammation.

[117] Dong XH, Ho MH, Liu B (2018). Role of *Porphyromonas gingivalis* outer membrane vesicles in oral mucosal transmission of HIV. Sci Rep.

[118] Fu Y, Wang M, Teng R, Li A (2025). Emerging roles of extracellular vesicles in the pathogenesis, diagnosis, and therapy of periodontitis. Biomedicines.

[119] Gegout PY, Mary B, Stutz C (2025). *Porphyromonas gingivalis* infection of oral keratinocytes drives the release of pro-inflammatory extracellular vesicles. Sci Rep.

[120] https://taggs.hhs.gov/Detail/AwardDetail?arg_AwardNum=R01AG088524&arg_ProgOfficeCode=102&utm_source=chatgpt.com.

[121] Zhu X, Zou A, Zhao M, Hou J, Xianyu Y (2025). Probiotic vesicles-implemented multifunctional nanotherapeutic approach for antibacterial, anti-inflammatory, and tissue regeneration in bacterial-infected oral ulcer healing. Adv Healthc Mater.

